# Preparation of a magnetic reduced-graphene oxide/tea waste composite for high-efficiency sorption of uranium

**DOI:** 10.1038/s41598-019-42697-7

**Published:** 2019-04-23

**Authors:** Aili Yang, Yukuan Zhu, Ping Li, C. P. Huang

**Affiliations:** 1 Institute of Materials, China Academy of Engineering Physics, Jiangyou, 621907 China; 2 Department of Environmental Engineering, University of Delaware, Newark, DE 19716 USA

**Keywords:** Pollution remediation, Environmental chemistry

## Abstract

The preparation and application of adsorptive materials with low cost and high-efficiency recovery of uranium from nuclear waste is necessary for the development of sustainable, clean energy resources and to avoid nuclear pollution. In this work, the capacity of tea waste and tea waste hybrids as inexpensive sorbents for uranium removal from water solutions was investigated. Composites of graphene oxide (GO) and tea waste (TW) exhibited a promising adsorption performance for uranium from aqueous solutions. The composites GOTW and magnetic rGO/Fe_3_O_4_/TW show high adsorption capacities (*Q*_m (TW)_ = 91.72 mg/g, *Q*_m (GOTW)_ = 111.61 mg/g and *Q*_m (rGO/Fe3O4/TW)_ = 104.95 mg/g) and removal rates (~99%) for U(VI). The equilibrium sorption of the adsorbents fitted well to the Langmuir model, and the sorption rate fitted well to a pseudo-second-order kinetic model. The thermodynamic parameters indicated that sorption was spontaneous and favourable. The prepared adsorbents were used for the removal of uranium from real water samples as well. The results revealed that GOTW and rGO/Fe_3_O_4_/TW can be used to remediate nuclear industrial effluent as a potential adsorbent.

## Introduction

To significantly reduce the emission of the greenhouse gas CO_2_ from burning fossil fuels and avoid air pollution-related deaths, nuclear power from fission currently accounts for ~10% of the global electricity supply^1^. Uranium is the main nuclear fuel of fission power reactors. However, uranium released into the environment and water results in serious hazards for various organisms, including humans, due to its high chemical toxicity, radioactivity and long half-life^2^. The US Environmental Protection Agency has established a maximum contaminant level for uranium in drinking water to be 30 μg/L^3^. Consequently, researchers have investigated methods for the efficient removal and recovery of uranium from aqueous solutions to protect the environment and control nuclides pollution. At present, various methods have been developed to address uranium in water, including membrane separation^4,5^, photoelectrochemical reduction^6^, biological treatment^7,8^, solvent extraction^9,10^, ion exchange^11,12^ and adsorption^13–15^. Adsorption is the most extensively used approach for uranium removal because of its high efficiency, low cost, simple operation and low production of secondary pollution.

Different types of adsorbents have been successfully used for uranium removal from aqueous solutions, including natural polymers^16,17^, inorganic compounds^18,19^, carbon nanomaterials^20,21^, metal-organic frameworks^22,23^ and biomass^24,25^. The economy of the adsorbents used is of great importance to reduce wastewater treatment costs. Many researchers have focused on the removal of various pollutants by low-cost adsorbents derived from agricultural wastes^26–28^. The re-utilization of agricultural wastes is increasingly becoming a significant concern due to their unique structures and outstanding physicochemical properties^29,30^.

Tea has been the most widely consumed beverage in many countries due to its reported positive health benefits^31,32^. The largest amount of production and consumption of tea is in China. The large amount of tea waste (TW) produced each year will inevitably result in severe environmental problems if not fully utilized. Tea leaves contain an abundance of active chemical constituents, including flavonoids, phenolic compounds, alkaloids and methylxanthines^33^. Additionally, TW has several salient advantages such as being inexpensive, easy to handle and having wide availability^34^. Therefore, it is necessary to take full advantage of TW to avoid environmental pollution and resource waste. TW has been widely investigated for the removal of various pollutants such as dyes^35^, oxytetracycline^36^, ethylene^37^, As and Ni^38^, Cu^39^, Cd^40^, Pb^41^ and Hg^0^ ^42^. To the best of our knowledge, few studies have applied TW as an adsorbent for the adsorption of uranium from aqueous solutions^43–46^, and the maximum sorption capacities (*Q*_m_) for uranium loading by TW obtained in these reports are low (<150 mg/g). Moreover, there is no literature available on the adsorption of uranium onto a magnetic reduced graphene oxide/Fe_3_O_4_/TW (rGO/Fe_3_O_4_/TW) composite adsorbent.

At present, the development and applications of graphene and its derivatives have been hot research topics in various fields such as electronic devices, electrochemical capacitors, catalysts and environmental remediation, thanks to their unusual properties^47^. Graphene oxide (GO) has been shown to be an excellent adsorbent for heavy metal ions (e.g., As^48^, Eu^49^, Cr^50^, Pb^51^, Cu^52^ and U^53^). GO has numerous active functional groups fixed on the surface including carboxyl, lactone, hydroxyl and phenolic hydroxyl^54^. TW is rich in cellulose, whose hydroxyl groups can be combined with GO to form an ester linkage, introducing carboxyl groups to TW^55^. In the present study, we prepared two novel composites, GOTW and rGO/Fe_3_O_4_/TW, and explored their application as an adsorbent for uranium(VI) from aqueous solutions. Moreover, the adsorptive performance for U(VI) was tested in a solution containing other cationic ions and in practical low-radioactive uranium-bearing nuclear wastewater. The results presented in this work reveal that the composite GOTW and magnetic rGO/Fe_3_O_4_/TW are promising adsorbents for the removal of uranium from nuclear industrial effluent.

## Results and Discussion

### Characterization of the products

**Figure 1:**
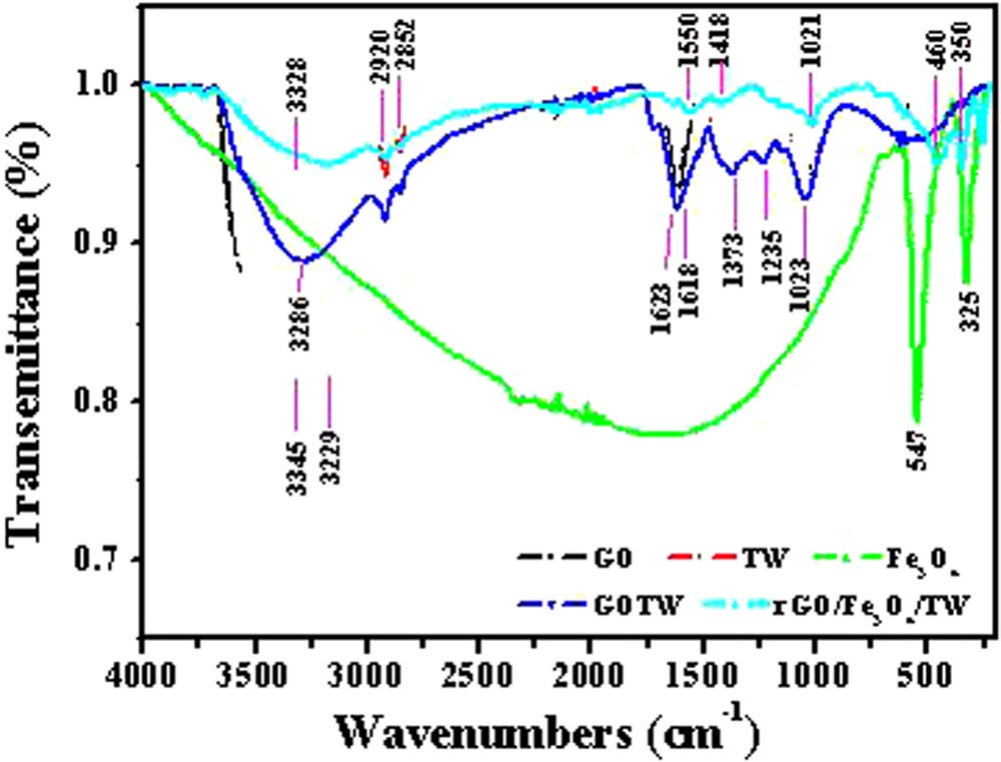
IR spectra of GO, Fe_3_O_4_, TW, GOTW and rGO/Fe_3_O_4_/TW.

The FT-IR spectra of GO, Fe_3_O_4_, TW, GOTW and rGO/Fe_3_O_4_/TW re shown in Fig. 1. The IR spectrum of GO was similar to that of GO in the reference^56^ and had characteristic peaks at 3345~3229, 1725, 1618, 1387, 1227 and 1061 cm^−1^, corresponding to the stretching vibrations of O-H, carbonyl and carboxyl C=O, aromatic C=C and H_2_O, carboxyl O=C-O, and alkoxy C-O-C stretching vibrational modes, respectively. In the IR spectrum of TW, the broad peaks at 3328 cm^−1^ could come from the -OH of absorbed H_2_O (3200–3500 cm^−1^) or N-H stretching. The double peaks at 2920 and 2852 cm^−1^ were attributed to aliphatic carbons. The strong peaks at 1627 and 1026 cm^−1^ indicated the carbonyl stretching of -COOH groups and stretching vibration of the C-O groups of polysaccharides, respectively. These characteristic peaks of the prepared TW were similar to that of tea wastes reported previously^43^. However, compared to that of GO, double peaks at 2920 and 2852 cm^−1^ attributed to aliphatic carbons appeared in the IR spectrum of GOTW, and the intensity of the characteristic peaks at 3286 cm^−1^ ascribed to O-H was significantly lower, which suggests that the composite GOTW was prepared successfully. In the IR spectrum of rGO/Fe_3_O_4_/TW, there were characteristic peaks at 460 cm^−1^ and 350 cm^−1^ belonging to the Fe-O stretching vibration^57^, and similar absorption peaks as TW, whereas the characteristic peaks assigned to the stretching vibrations of O-H, carbonyl and carboxyl C=O, aromatic C=C and H_2_O, carboxyl O=C-O, and alkoxy C-O-C disappeared. Moreover, the stretching vibrations of C=C and epoxy C-O at 1550 and 1014 cm^−1^ of rGO functional groups were present in the IR spectrum of rGO/Fe_3_O_4_/TW^58^. These results implied that GO is reduced to rGO and that, simultaneously, Fe(II) is oxidized to Fe(III). The results showed that the magnetic composite rGO/Fe_3_O_4_/TW was prepared successfully.

**Figure 2:**
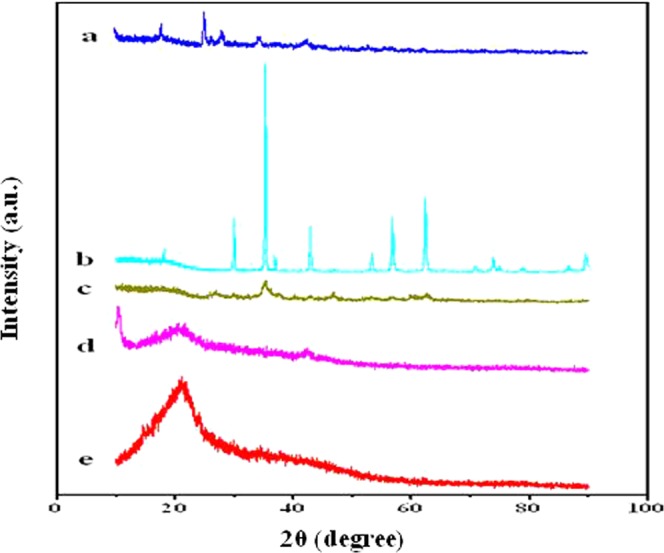
XRD patterns of GO (**a**), Fe_3_O_4_ (**b**), rGO/Fe_3_O_4_/TW (**c**), GOTW (**d**) and TW (**e**).

The XRD patterns of the crystal phase of the prepared GO (a), Fe_3_O_4_ (b), rGO/Fe_3_O_4_/TW (c), GOTW (d) and TW (e) are presented in Fig. 2. No obvious diffraction peaks at 2θ=10° and 42° attributed to the crystal planes of GO were observed, confirming the reduction of GO to rGO. The XRD pattern of TW (Fig. 2e) was consistent with that previously reported for tea^59^. Compared to those of GO and TW, differences in the peak intensities in the XRD pattern of GOTW (Fig. 2d) indicated that TW was successfully attached to the surface of GO. The XRD peaks at 2θ values of approximately 29.94°, 35.30°, 42.98°, 53.38°, 56.84°, 62.46° and 74.54° of the purchased Fe_3_O_4_ are shown in Fig. 2b. The diffraction peaks of the composite rGO/Fe_3_O_4_/TW (Fig. 2c) are consistent with those of Fe_3_O_4_, but the peak intensities were significantly lower with the addition of rGO and TW.

**Figure 3:**
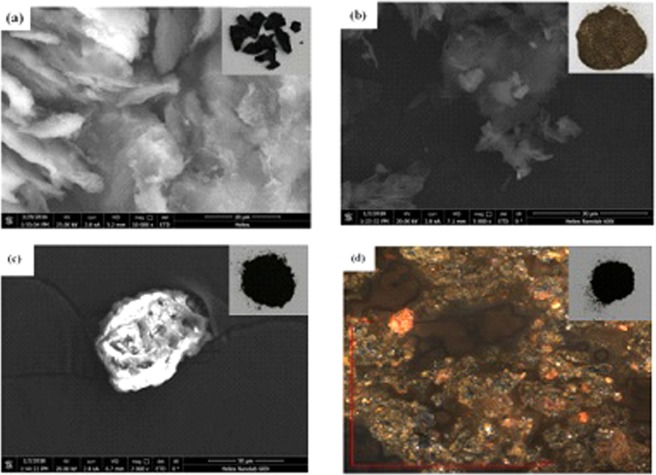
SEM images of GO (**a**), TW (**b**), GOTW (**c**) and AFM image of rGO/Fe_3_O_4_/TW (**d**). Insets are optical digital photos of GO, TW, GOTW and rGO/Fe_3_O_4_/TW.

The morphologies of GO, TW and GOTW were observed by SEM. The morphology of rGO/Fe_3_O_4_/TW was analysed by AFM because of its magnetic properties. Figure 3a shows that GO exhibited a wrinkled lamellar structure, and the sheets of GO stacked together due to strong inter-planar interactions. As shown in Fig. 3b, an irregular layered structure with a smooth surface without pores was observed in the SEM image of TW. However, the surface of GOTW (Fig. 3c) presented many tiny pores which could be propitious to adsorb heavy metal ions^60^. In the AFM image of rGO/Fe_3_O_4_/TW (Fig. 3d) we observed that many Fe_3_O_4_ particles were attached to the surface of GOTW. Moreover, as seen in the insets of Fig. 3, a significant difference in the macroscopic morphology of the prepared sorbents was observed.

**Figure 4:**
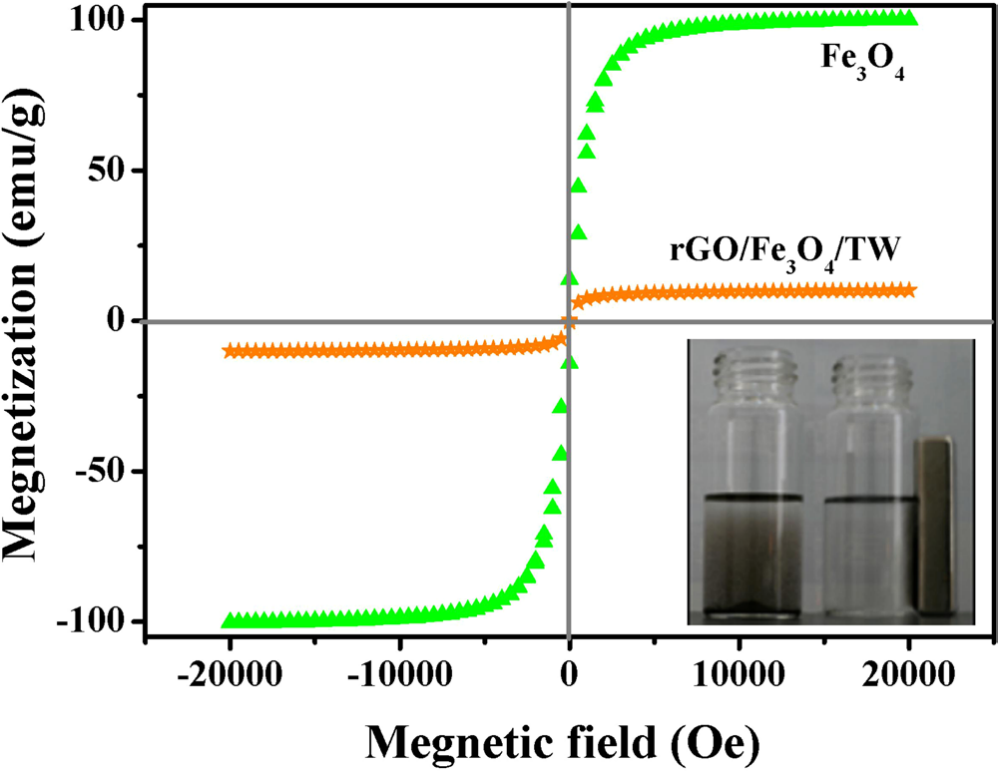
Magnetization curve of Fe_3_O_4_ and rGO/Fe_3_O_4_/TW. Inset is the separation application from the solution of rGO/Fe_3_O_4_/TW by an external magnetic field.

Magnetic analysis of Fe_3_O_4_ and rGO/Fe_3_O_4_/TW using a vibrating sample magnetometer (VSM) is shown in Fig. 4. The magnetization saturation values for Fe_3_O_4_ and rGO/Fe_3_O_4_/TW were 100 and 10 emu/g, respectively. A nonlinear, reversible magnetization curve with no hysteresis exhibited characteristic super-paramagnetic behaviour. The reduced saturation magnetization was mainly due to the presence of diamagnetic GOTW surrounding the Fe_3_O_4_ cores. However, as seen in the inset of Fig. 4, the composite rGO/Fe_3_O_4_/TW can be completely separated from the solution by a conventional magnet.

### XPS analysis of the samples

**Figure 5:**
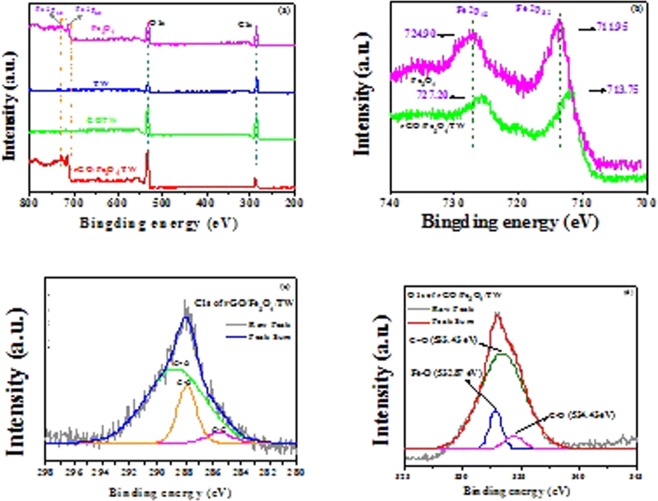
XPS survey spectra of Fe_3_O_4_, TW, GOTW and rGO/Fe_3_O_4_/TW (**a**), the high-resolution Fe 2p spectra of Fe_3_O_4_ and rGO/Fe_3_O_4_/TW (**b**), the high-resolution C1s spectra of TW and rGO/Fe_3_O_4_/TW (**c**) and the high-resolution O1s spectra of rGO/Fe_3_O_4_/TW and Fe_3_O_4_ (**d**).

The chemical composition of Fe_3_O_4_, TW, GOTW and rGO/Fe_3_O_4_/TW was investigated by XPS. The XPS survey spectra of rGO/Fe_3_O_4_/TW (Fig. 5a) showed evident characteristic peaks at approximately 289, 557, 715 and 729 eV, which were attributed to C1s, O1s and Fe2p, respectively. In the high-resolution spectrum of Fe 2p (Fig. 5b), the peaks of Fe 2p_3/2_ and Fe 2p_1/2_ were located at 713.75 and 727.20 eV, respectively, indicating the presence of Fe_3_O_4_ in the composite rGO/Fe_3_O_4_/TW. The C1s and O1s XPS peaks of TW and rGO/Fe_3_O_4_/TW were deconvoluted (Fig. 5c,d). Figure 5c shows the C1s spectra with three deconvoluted peaks at 283.8, 285.1 and 287.5 eV associated with C–C, C–O and C=O bonds, respectively. Figure 5(d) shows the O1s spectrum, and the three peaks at 529.6, 531.2 and 532.7 eV belong to Fe–O, C=O and C–O, respectively. The fit results are presented in Table 1. The oxygen-containing groups C-O and C=O were significantly more abundant after combination, implying that TW was successfully loaded onto the surface of GO.

**Table 1:** Curve fitting results of XPS C1s spectra of TW and rGO/Fe_3_O_4_/TW.

Peak	rGO/Fe_3_O_4_/TW		TW	
	Position/eV	%	Position/eV	%
C–C/C=C	285.60	4.07	284.99	78.53
C–O	287.88	18.76	286.81	2.93
C=O	288.74	77.17	287.27	18.54

**Figure 6:**
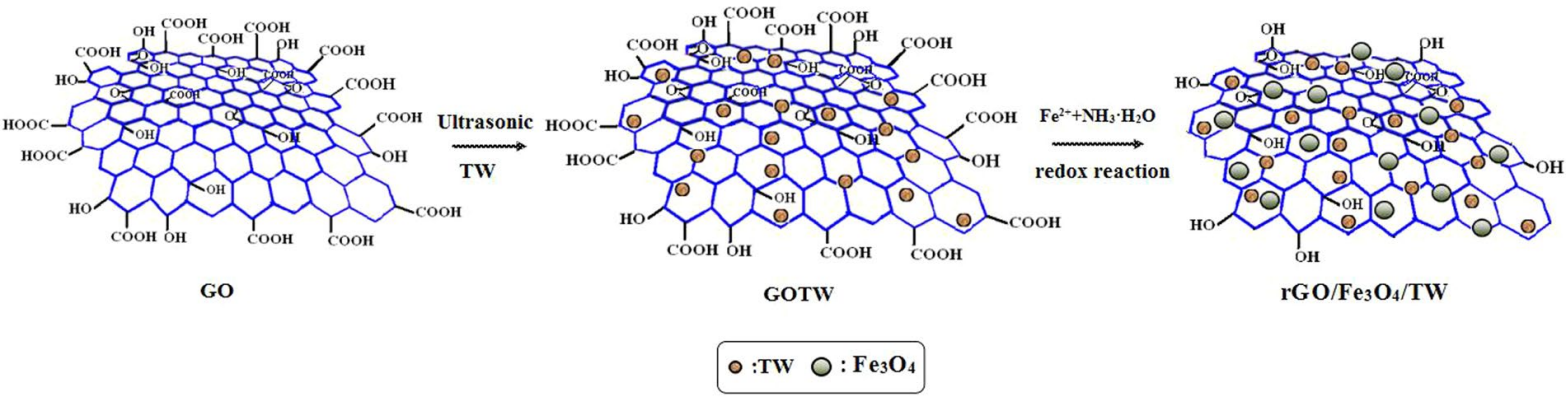
Schematic diagram of a possible formation mechanism of rGO/Fe_3_O_4_/TW.

Based on the results described above, a possible reaction mechanism for the composite rGO/Fe_3_O_4_/TW is illustrated in Fig. 6. First, GO combines with TW to form GOTW, and then Fe^2+^ ions react with GOTW by a redox reaction to form rGOTW as shown in Eq. (2) in which GO is as an oxidant. Because the hydrolysis of Fe^3+^ results in the formation of Fe(OOH), as shown in Eq. (3), the composite rGO/Fe_3_O_4_/TW was obtained after the addition of ammonia solution, as shown in Eq. (4).

1 $${\rm{GO}}+{\rm{TW}}\to {\rm{GOTW}}$$

2 $${\rm{GOTW}}+{{\rm{Fe}}}^{2+}\,\mathop{\longrightarrow }\limits^{{\rm{redox}}\,{\rm{reaction}}}\,{{\rm{Fe}}}^{3+}+{\rm{rGOTW}}$$

3 $${{\rm{Fe}}}^{3+}+{{\rm{H}}}_{{\rm{2}}}{\rm{O}}\to {\rm{Fe}}({\rm{OOH}})$$

4 $${\rm{rGOTW}}\cdot {{\rm{Fe}}}^{n+}({\rm{n}}=2,3)+\mathrm{Fe}({\rm{OOH}}){+\mathrm{NH}}_{{\rm{3}}}\cdot {{\rm{H}}}_{{\rm{2}}}{\rm{O}}\to {\mathrm{rGO}/\mathrm{Fe}}_{{\rm{3}}}{{\rm{O}}}_{{\rm{4}}}/\mathrm{TW}$$

### Influence of pH on adsorption

**Figure 7:**
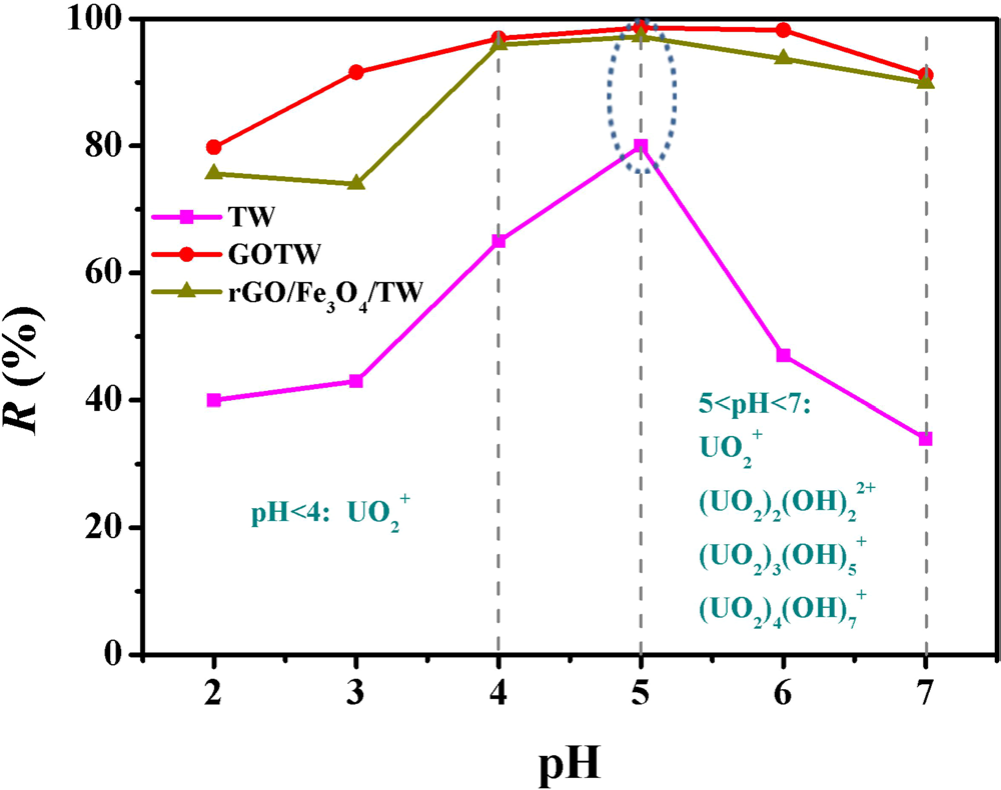
Effect of pH on the U(VI) adsorption by TW, GOTW and rGO/Fe_3_O_4_/TW. *C*_(U)initial_ = 10 mg/L, *C*_sorbent_ = 0.5 g/L, contact time = 30 min, and *T* = 298 K.

The solution pH affects the speciation of uranium in solution and significantly influences the uranium adsorption process. The effect of pH on the adsorption of uranium (VI) by the synthesized adsorbents is presented in Fig. 7. The results showed a substantial impact of pH on uranium adsorption. The adsorption of U(VI) on TW, GOTW and rGO/Fe_3_O_4_/TW significantly increased with increasing pH from 2.0 to 5.0. At pH = 5, the removal rate of uranium of TW, GOTW and rGO/Fe_3_O_4_/TW reached the highest value. At pH < 4.0, uranium exists mainly in the form of UO_2_^2+^. The competition between H_3_O^+^ and UO_2_^2+^ for binding sites on the adsorbent surface results in a low sorption efficiency^61^. At pH = 5.0~7.0, the prominent species of uranium in the solution are UO_2_^+^, UO_2_(OH)^+^, (UO_2_)_2_(OH)_2_^2+^, (UO_2_)_3_(OH)_5_^+^ and (UO_2_)_4_(OH)_7_^+^ ^62^. As seen in Fig. 7, the removal rate of uranium significantly increases at pH > 5.0, which can be attributed to electrostatic interactions of these complex uranium ions with negatively charged groups on the surface of TW, GOTW and rGO/Fe_3_O_4_/TW. The sorption behaviour of UO_2_^2+^ on GOTW and rGO/Fe_3_O_4_/TW at pH 2.0~7.0 is similar to that reported by Wang *et al*.^13^. Additionally, the sorption efficiency of uranium by GOTW and rGO/Fe_3_O_4_/TW was higher than that for TW, indicating that the composite of GO and TW had more efficient adsorption of uranium. Consequently, the optimum pH for U(VI) adsorption by TW, GOTW and rGO/Fe_3_O_4_/TW was 5.0.

### Influence of contact time and kinetic study

**Figure 8:**
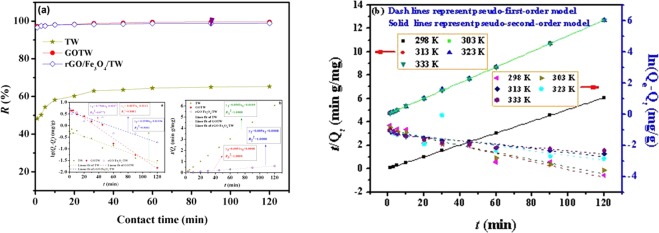
Influence of contact time on U(VI) sorption and kinetic fit of TW, GOTW and rGO/Fe_3_O_4_/TW at 298 K (**a**) and kinetic fit of rGO/Fe_3_O_4_/TW at different tempetature (298 K–333 K) (**b**). pH = 5.0, *C*_(U)initial_ = 10 mg/L, *C*_sorbent_ = 0.5 g/L.

The adsorption dynamics data^63,64^ were analysed based on Eqs (5) and (6). Figure 8(a) presents the time-dependent adsorption over a contact time ranging from 1 to 120 min of U(VI) by TW, GOTW and rGO/Fe_3_O_4_/TW. As seen in Fig. 8(a), the removal rate of the prepared adsorbents exceeded 96% within 1 min, and the adsorption equilibrium time was 60 min. Adsorption kinetic data of the pseudo-first-order and pseudo-second-order model at different temperature are given in Table 2. The correlation coefficient of pseudo-second-order model (*R*^2^ = 0.9999 and 1.0000) was superior to the pseudo-first-order model, which indicated that the adsorption of U(VI) onto TW, GOTW and rGO/Fe_3_O_4_/TW fitted the pseudo-second-order model better. The fit results demonstrated that a chemical reaction played a significant role in the rate-controlling steps. The surface functional groups of the prepared adsorbents might form strong electrostatic and chemical interactions with U(VI) ions^65^. Moreover, the kinetic model fits results and parameters of rGO/Fe_3_O_4_/TW at different temperature are shown in Fig. 8(b) and Table 2, respectively. The result shows that temperature has no evident effect on the adsorption rate of rGO/Fe_3_O_4_/TW.

5 $${\rm{lg}}({Q}_{e}-{Q}_{t})=\,{\rm{lg}}\,{Q}_{e}-\frac{{k}_{1}}{2.303}t$$

6 $$\frac{t}{Q}=\frac{1}{{k}_{2}{{Q}_{e}}^{2}}+\frac{t}{{Q}_{e}}$$

where *k*_1_ (min^−1^) is the Lagergren rate constant of adsorption, and *k*_2_ (g/(mg·min)) is the rate constant of pseudo-second-order adsorption.

**Table 2:** Parameters of pseudo-first-order and pseudo-second-order kinetic models for U adsorption by TW, GOTW and rGO/Fe_3_O_4_/TW at different temperature.

Sorbents	Tempetature (K)	Pseudo-first-order model			Pseudo-second-order model		
		*Q*_*e*_ (mg/g)	*k*_1_ (min^−1^)	*R* ^2^	*Q*_*e*_ (mg/g)	*k*_2_ (g/(mg·min))	*R* ^2^
TW	298	1.026	0.0002	0.7400	19.88	0.2321	1.0000
GOTW	298	5.677	0.0500	0.9796	20.00	0.3135	1.0000
rGO/Fe_3_O_4_/TW	298	0.104	0.0594	0.9328	19.80	0.3985	1.0000
	303	0.082	0.0530	0.9510	19.80	0.4305	1.0000
	313	0.061	0.0290	0.9335	19.84	0.5522	1.0000
	323	0.083	0.0364	0.5729	19.92	0.2083	0.9999
	333	0.051	0.0244	0.8482	19.92	0.7412	1.0000

### Adsorption isotherm

**Figure 9:**
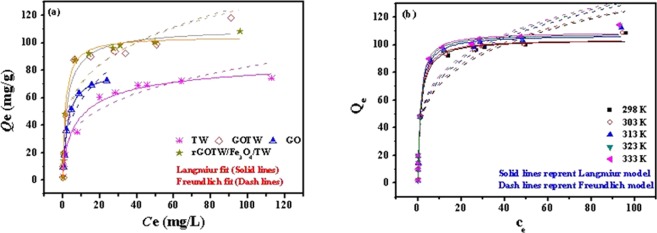
Langmuir and Freundlich isotherm models fit of GO, TW, GOTW and rGO/Fe_3_O_4_/TW at 298 K (**a**) and the isotherm models fit of rGO/Fe_3_O_4_/TW at different temperature (298 K-333 K) (**b**). pH = 5.0, *C*_(U)initial_ = 5-150 mg/L, *C*_sorbent_ = 0.5 g/L, contact time = 24 h.

The Langmuir and Freundlich isotherm models are expressed in Eqs (7) and (8), respectively^66^. The fit results of Langmuir and Freundlich isotherm models of GO, TW, GOTW and rGO/Fe_3_O_4_/TW at 298 K are shown in Fig. 9(a). The Langmuir and Freundlich isotherm models of rGO/Fe_3_O_4_/TW at different temperature are shown in Fig. 9(b). The isotherm parameters calculated from fitting processes at different temperature are listed in Table 3. The Langmuir equation of the adsorbents fitted the experimental data well, with a higher correlation coefficient (*R*2).

7 $${Q}_{e}=\frac{{Q}_{m}\cdot {K}_{L}\cdot {C}_{e}}{1+{K}_{L}{C}_{e}}$$

8 $${Q}_{e}={K}_{F}\cdot {{C}_{e}}^{1/n}$$

where *Q*_*e*_ (mg/g) is the equilibrium adsorption capacity, *C*_e_ (mg/L) is the uranium concentration at equilibrium, *Q*_*m*_ (mg/g) is the maximum adsorption capacity, *K*_L_ (L/mg) and *K*_F_ (mg^1−*n*^ L^n^/g) are the Langmuir and Freundlich constants, respectively, and *n* is the Freundlich adsorption exponent.

**Table 3:** Langmuir, Freundlich and D-R model parameters for uranium adsorption on GO, TW, GOTW and rGO/Fe_3_O_4_/TW at different temperature.

Sorbents	Tempetature (K)	Langmuir model			Freundlich model			D–R model		
		*Q*_m_ (mg/g)	*k*_L_ (L/mg)	*R* ^2^	*n*	*k*_F_ (mg^1−*n*^ L^n^/g)	*R* ^2^	*Q*_m_ (mg/g)	*β* (mol^2^/J^2^)	*R* ^2^
GO	298	79.108	0.3856	0.9901	2.89	26.3486	0.9134	59.369	10^−7^	0.7866
TW	298	91.719	0.2151	0.9797	3.42	21.5161	0.9320	56.165	10^−7^	0.9278
GOTW	298	111.614	0.4828	0.9682	3.84	38.4609	0.9006	78.830	10^−8^	0.9277
rGO/Fe_3_O_4_/TW	298	103.840	0.7519	0.9892	4.07	40.2672	0.8838	72.341	5 × 10^−8^	0.8664
	303	104.130	0.8218	0.9899	4.13	41.6600	0.8808	72.756	4 × 10^−8^	0.8664
	313	107.210	0.8096	0.9912	4.17	42.7600	0.8887	73.821	4 × 10^−8^	0.8632
	323	108.220	0.8689	0.9924	4.20	44.0200	0.8911	73.056	3 × 10^−8^	0.8521
	333	109.160	0.9188	0.9934	4.27	45.2600	0.8899	73.831	3 × 10^−8^	0.8557

Moreover, the Dubinin–Radushkevich (D–R) isotherm is applied to estimate the U(VI) adsorption behaviour (chemical or physical) onto the sorbent. The D–R equation^67^ is written as follow:

9 $$\mathrm{ln}\,{Q}_{e}=\,\mathrm{ln}\,{Q}_{m}-\beta {\varepsilon }^{2}$$

10 $$\varepsilon =RT\,\mathrm{ln}(1+\frac{1}{{C}_{e}})$$

where β (mol^2^/J^2^) is activity coefficient depending on the mean free energy of adsorption and ε is the Polanyi potential (J/mol). R and T represent the gas constant (8.314 J/mol K) and absolute temperature (K), respectively.

The D–R isotherm fit parameters of GO, TW, GOTW and rGO/Fe_3_O_4_/TW at different temperature are listed in Table 3. The D–R isotherm usually used the mean free energy *E* (kJ/mol) to assess the type of adsorption mechanism. The E value can be calculated according to Eq. (11). A value higher than 8 kJ/mol is considered to indicate chemical adsorption while it is less than 8 kJ/mol for the physical adsorption^68^. Seen from Table 3, the calculated *E* value of GO, TW, GOTW and rGO/Fe_3_O_4_/TW at 289 K was 2.236 kJ/mol, 2.236 kJ/mol, 2.887 kJ/mol and 3.162 kJ/mol, respectively which indicated that physical adsorption occurs for U(VI) onto GO, TW, GOTW and rGO/Fe_3_O_4_/TW.

11 $$E=\frac{1}{\sqrt{2\beta }}$$

A comparison of the *Q*_m_ of different adsorbents is presented in Table 4. Table 4 shows that the *Q*_m_ of the prepared rGO/Fe_3_O_4_/TW indicated a promising adsorbent for the treatment of uranium-bearing wastewater.

**Table 4:** Comparison of *Q*_m_ of as-obtained adsorbents in this study with the reported tea wastes for U(VI) adsorption.

Sorbents	pH	c/V (g/L)	*Q*_m_ (mg/g)
Tea wastes^43^	4	1.2	29.41
Tea waste^45^	6	0.2	142.21
Pyrolyzed tea wastes^46^	3	—	59.50
Green tea^69^	4~6	5	8.12
Tea waste^70^	6	—	43.19
Black tea scrap^71^	5	4	62.51
TW (This work)^70,71^	5	0.5	91.72
GOTW (This work)	5	0.5	111.61
rGO/Fe_3_O_4_/TW (This work)	5	0.5	104.95

### Effect of temperature and adsorption thermodynamics

**Figure 10:**
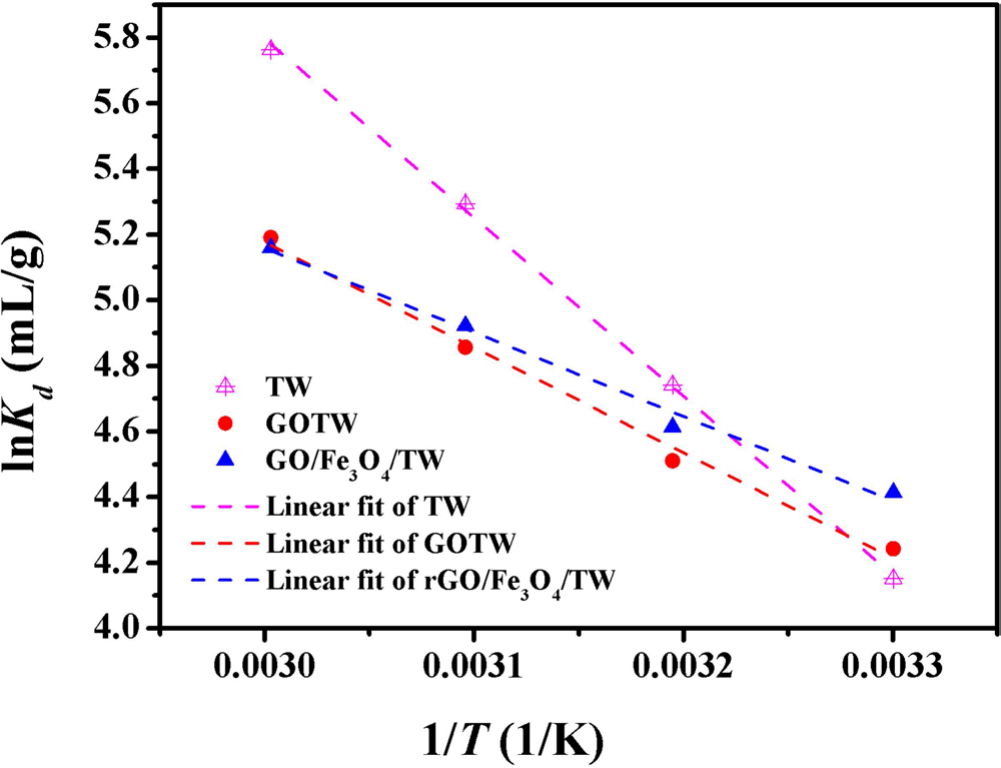
Plots of ln*K*_*d*_ versus 1/*T* for U(VI) adsorption onto TW, GOTW and rGO/Fe_3_O_4_/TW. pH = 5.0, *C*_(U)initial_ = 10 mg/L, *C*_sorbent_ = 0.5 g/L, *T* = 303 K, 313 K, 323 K and 333 K, and contact time = 24 h.

The enthalpy (Δ*H*^0^), entropy (Δ*S*^0^) and standard free energy Δ*G*^0^ from 303 K to 333 K in the adsorption process were calculated from the slope and intercept of the linear line of ln*K*_*d*_ versus 1/*T* using Eqs (12)~(14)^15^. The thermodynamic parameters and the plots of ln*K*_d_ versus 1/*T* onto TW, GOTW and rGO/Fe_3_O_4_/TW are shown in Table 5 and Fig. 10, respectively. The negative value of Δ*H*^0^ indicated that the adsorption reaction was endothermic. The positive Δ*S*^0^ and negative Δ*G*^0^ sugges that the adsorption process was spontaneous.

12 $${K}_{d}=\frac{{c}_{ad}}{{c}_{e}}$$

13 $$\mathrm{ln}\,{K}_{d}=-\,\frac{{\rm{\Delta }}{H}^{0}}{RT}+\frac{{\rm{\Delta }}{S}^{0}}{R}$$

14 $${\rm{\Delta }}{G}^{0}={\rm{\Delta }}{H}^{0}-T{\rm{\Delta }}{S}^{0}$$

where *K*_*d*_ (mL/g) is the distribution coefficient of U(VI), *c*_ad_ (mg/L) is the concentration of metal ions on the adsorbent at equilibrium, *c*_e_ (mg/L) is the equilibrium concentration of metal ions in solution.

**Table 5:** Thermodynamic parameters of U(VI) adsorption on TW, GOTW and rGO/Fe_3_O_4_/TW.

Sorbents	Δ*H*^0^ (kJ/mol)	Δ*S*^0^ (J/(mol·k))	Δ*G*^0^ (kJ/mol)			
			303 K	313 K	323 K	333 K
TW	45.24	183.91	−10.48	−12.32	−14.16	−16.00
GOTW	26.71	123.16	−10.61	−11.83	−13.07	−14.30
rGO/Fe_3_O_4_/TW	21.31	106.83	−11.06	−12.13	−13.20	−14.26

### Real wastewater sample analysis

**Figure 11:**
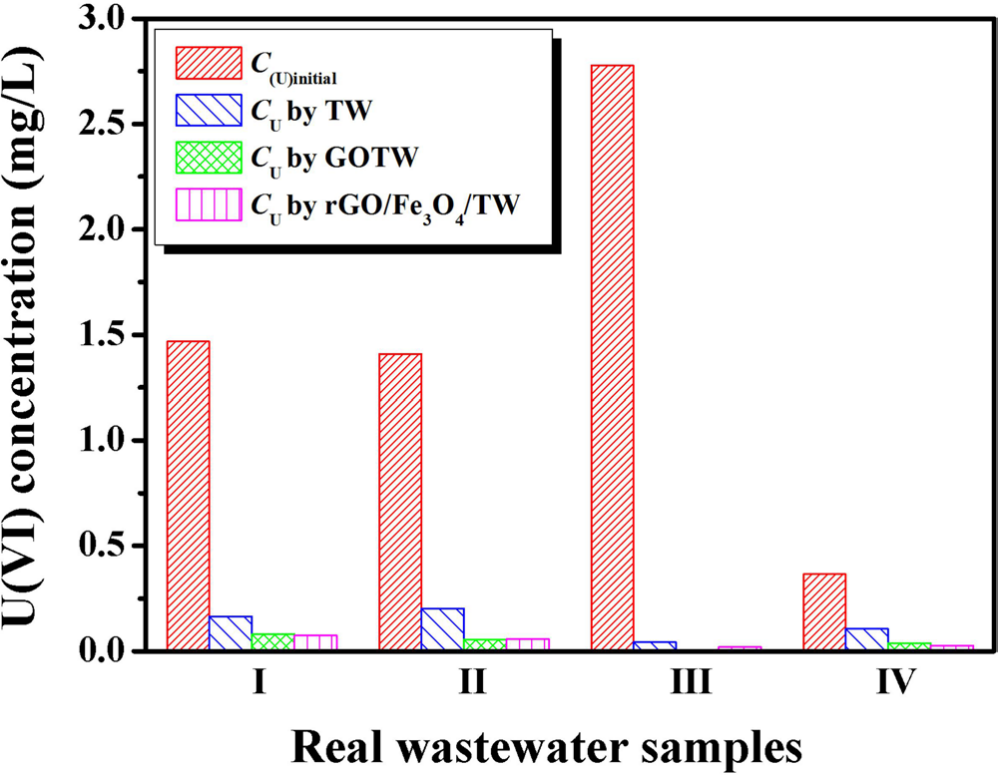
Column chart of adsorption efficiency of TW, GOTW and rGO/Fe_3_O_4_/TW for real uranium-bearing nuclear waste effluents. pH = 5.0, *C*_sorbent_ = 0.5 g/L, contact time = 1 h, and *T* = 298 K.

To evaluate the applicability of the prepared adsorbents in this study for real uranium-bearing wastewater samples, the removal efficiency of U(VI) from four different batches of wastewater samples under the optimum adsorption conditions was investigated. The measured parameters of real nuclear wastewater and adsorption experiment results are presented in Table 6 and Fig. 11, respectively. As shown in Fig. 11, co-existing ions had no effect on the removal efficiency of GOTW and rGO/Fe_3_O_4_/TW for uranium, and they can to be applied in the treatment of uranium-containing nuclear waste effluents as potential adsorbents.

**Table 6:** Measured parameter values of real uranium-bearing wastewater samples.

Wastewater parameters	Measured parameter values	Wastewater parameters	Measured parameter values
pH	7.0~8.0	U	0.3~3.0 mg/L
Al	≤0.05 mg/L	Ca	0~5.00 mg/L
B	≤0.05 mg/L	Be	≤0.05 mg/L
Cu	0~0.04 mg/L	Fe	≤0.34 mg/L
Mn	0~0.04 mg/L	Mg	0~1.38 mg/L
Si	0~2.71 mg/L	Mo	≤0.05 mg/L
Ni	0~0.02 mg/L

## Materials and Methods

### Materials

Stock solutions of uranium (5~150 mg/L) were prepared by dissolving UO_2_(NO_3_)_2_·6H_2_O (Xi’an Dingtian Chemical Reagent Co.) in deionized water (DW) and acidifying with a small amount of concentrated HNO_3_. The green tea used in this work originated from Sichuan Pingwu in China. All reagents used were of analytical grade and were used without further purification. DW was used throughout the experiments.

### Preparation of tea wastes (TW)

Green tea was washed with DW several times to remove all dirt substances. It was then boiled in DW at 80 °C for 1 h to remove coloured and soluble components, and then washed with DW until virtually colourless. The obtained TW was dried in an oven at 100 °C for 24 h. Finally, the products were crushed to powder in a pulveriser^72^.

### Preparation of the composite GOTW

GO was prepared from natural graphite by the modified Hummers method^73^. GOTW was prepared using GO and TW under ultrasonic treatment. Briefly, the mixture of GO (0.5 g) and TW (0.5 g) was dispersed in 100 ml DW under ultrasonication for 3 h. The obtained GOTW was centrifuged and washed with DW and ethanol. Finally, GOTW was dried at 50 °C under vacuum.

### Preparation of the magnetic composite rGO/Fe_3_O_4_/TW

The rGO/Fe_3_O_4_/TW composite was synthesized using a previously reported method^74^. First, a mixture of GO (0.25 g) and TW (0.25 g) was dispersed in 100 ml DW under ultrasonic treatment for 3 h. Then, 25% dilute ammonia solution was added drop-wise to the solution until the pH reached 11. Then, 1.25 g of FeCl_2_·4H_2_O was added very slowly to the mixture with continuous magnetic stirring. After stirring for 3 h, black rGO/Fe_3_O_4_/TW product was obtained by filtration and washed with DW and ethanol. Finally, rGO/Fe_3_O_4_/TW was dried at 50 °C under vacuum.

### Characterization of the products

Fourier transform infrared (FTIR) spectra of as-prepared adsorbents were obtained using an FTIR spectrometer (Bruker VERTEX 70, Germany). The crystal phases of the samples were characterized by X-ray diffractometer (XRD) (2700 model, China). The surface morphology of the products was determined using a scanning electron microscope (SEM; FEI Helios 600i, USA). The magnetic measurements of Fe_3_O_4_ and rGO/Fe_3_O_4_/TW were conducted at 300 k under a varying magnetic field (PPMS-9 ECII, USA Quantum Design Co.). X-ray photoelectron spectroscopy (XPS) was studied using an ESCALAB 250 X-ray photoelectron spectroscopy (Thermo fisher, USA).

### Adsorption experiments

The influence of pH, contact time, initial uranium concentration, and temperature on the removal efficiency of uranium were investigated. The solution pH was adjusted using NaOH and HCl. The prepared adsorbent was added to 20 mL U(VI) solution and shaken in a shaker (Kangshi, China). After filtration, residual uranium concentrations were measured by a micro-quantity uranium analyser (MUA model, China). The removal rate *R* (%) and the adsorption capacity of U(VI) *Q* (mg/g) were calculated according to Eqs (15) and (16), respectively:

15 $$R( \% )=\frac{{c}_{0}-{c}_{t}}{{c}_{0}}\times 100$$

16 $$Q(mg/g)=\frac{({c}_{0}-{c}_{t})}{W}\times V$$

where *c*_0_ (mg/L) is the initial uranium (VI) concentration, *c*_*t*_ (mg/L) is the uranium concentration at time *t*, *V* (L) is the solution volume and *W* (g) stands for the weight of adsorbent.

## Conclusion

In summary, four adsorbents GO, TW, GOTW and rGO/Fe_3_O_4_/TW were fabricated for the adsorption of uranium from aqueous solutions. The adsorption data of U(VI) by TW, GOTW and rGO/Fe_3_O_4_/TW were consistent with the Langmuir isotherm model and pseudo-second-order kinetics. The composites GOTW and rGO/Fe_3_O_4_/TW exhibited higher adsorption capacities and faster adsorption kinetics than did GO and TW. The results showed that GOTW and rGO/Fe_3_O_4_/TW could be utilized effectively as promising sorbents to removal uranium from real multi-component uranium-containing nuclear waste effluents. Furthermore, due to the advantageous magnetic properties, rGO/Fe_3_O_4_/TW can be easily separated from aqueous solutions, thus enhancing post-treatment efficiency for further practical applications.
